# Enhancing Cognition in Older Adults with Mild Cognitive Impairment through High-Intensity Functional Training: A Single-Blind Randomized Controlled Trial

**DOI:** 10.3390/jcm12124049

**Published:** 2023-06-14

**Authors:** Yulieth Rivas-Campo, Agustín Aibar-Almazán, Carlos Rodríguez-López, Diego Fernando Afanador-Restrepo, Patricia Alexandra García-Garro, Yolanda Castellote-Caballero, Alexander Achalandabaso-Ochoa, Fidel Hita-Contreras

**Affiliations:** 1 Faculty of Human and Social Sciences, University of San Buenaventura-Cali, Santiago de Cali 760016, Colombia; yrivasc@usbcali.edu.co; 2 Department of Health Sciences, Faculty of Health Sciences, University of Jaén, 23071 Jaén, Spain; mycastel@ujaen.es (Y.C.-C.); aaochoa@ujaen.es (A.A.-O.); fhita@ujaen.es (F.H.-C.); 3 Lecturer University Schools Gimbernat, University of Cantabria, 39005 Santander, Spain; carlinhosdin@gmail.com; 4 Faculty of Health Sciences and Sport, University Foundation of the Área Andina–Pereira, Pereira 660004, Colombia; dafanador4@areandina.edu.co; 5 Faculty of Distance and Virtual Education, Antonio José Camacho University Institution, Santiago de Cali 760016, Colombia; palexandragar-cia@admon.uniajc.edu.co

**Keywords:** cognition, physical activity, verbal fluency, processing speed, mild cognitive impairment

## Abstract

Physical exercise is a very promising non-pharmacological approach to prevent or reduce the cognitive decline that occurs in people aged 60 years or older. The objective of this study was to determine the effect of a high-intensity intervallic functional training (HIFT) program on cognitive functions in an elderly Colombian population with mild cognitive impairment. A controlled clinical trial was developed with a sample of 132 men and women aged >65 years, linked to geriatric care institutions, which were systematically blind randomized. The intervention group (IG) received a 3-month HIFT program (*n* = 64) and the control group (CG) (*n* = 68) received general physical activity recommendations and practiced manual activities. The outcome variables addressed cognition (MoCA), attention (TMTA), executive functions (TMTB), verbal fluency (VFAT test), processing speed (Digit Symbol Substitution Test-DSST), selective attention and concentration (d2 test). After the analysis, improvement was found in the IG with significant differences with respect to the CG in the level of cognitive impairment (MoCA), attention (TMTA), verbal fluency and concentration (*p* < 0.001). Executive functions (TMTB) showed differences in both groups, being slightly higher in the IG (*p* = 0.037). However, no statistically significant results were found for selective attention (*p* = 0.55) or processing speed (*p* = 0.24). The multiple analysis of covariance (MANCOVA) showed the influence of the education level on all cognition assessments (*p* = 0.026); when adjusting for sociodemographic variables, the influence of the intervention remained significant (*p* < 0.001). This study empirically validates that the implementation of a HIFT program has a positive effect on cognitive functions in elderly people with mild cognitive impairment. Therefore, professionals specialized in the care of this population could consider including functional training programs as an essential part of their therapeutic approaches. The distinctive features of this program, such as its emphasis on functional training and high intensity, appear to be relevant for stimulating cognitive health in the geriatric population.

## 1. Introduction

Cognition, defined as the ability to learn, remember, solve problems and appropriately use previously stored information, is one of the most important factors in healthy aging [1]. Multiple complex structures are strongly affected by aging, generating neuroanatomical and neurophysiological changes that impact cognition [2]. One of the pathologies that are associated with cognition as a result of aging is mild cognitive impairment (MCI).

MCI is considered the transition point between the cognition problems expected during aging and dementia [3]. Although it is a relatively common disease, that affects between six and fifty million people worldwide with a prevalence that tends to double every five years [4], no pharmacological treatment exists yet that can prevent or cure it [5]. Considering that the decline in cognition has been multiple times negatively associated with the practice of physical activity [6,7] and that the prevalence of sedentary lifestyles along with life expectancy is increasing [8,9], it is logical to think that this disease will become a public health problem in the medium term.

Hence, many researchers have focused their attention on this pathology, seeking cost-effective treatments that can help to reduce the economic burden on the health systems that this disease represents. Among the possible interventions, physical exercise is highlighted for its effects on blood flow [10], oxidative stress [11] and brain volume [12], which could mitigate the impact of aging and thus delay the onset of diseases that lead to cognitive impairment.

Several authors have investigated the effects of physical activity on variables associated with cognition, such as verbal fluency, processing speed, and selective attention, and have observed favorable results in the older adult population [13,14]. In recent years, there has been an increasing recognition that these cognitive variables can serve as predictors of cognitive impairment, enabling early diagnosis and a more accurate prognosis [15]. Consequently, it is widely considered that exercise practice generates positive effects on cognition in older adults with MCI.

One training method that has gained particular attention is the high-intensity and short-duration training, known as High-Intensity Interval Training (HIIT) or its variant High-Intensity Functional Training (HIFT), which, unlike HIIT, focuses on the execution of multi-joint exercises with a functional approach that can be adapted to any level of physical condition [16]. HIFT programs have already been shown to be useful as a means of improving gait speed, strength, quality of life, muscle function and metabolic performance [17,18,19], among others in different populations, but their effects on cognition in older adults with MCI are still unclear [20]. Therefore, this study seeks to determine the effects of HIFT on different variables associated with cognition in Colombian older adults with MCI.

## 2. Materials and Methods

### 2.1. Study Design

This randomized controlled clinical trial (NCT04638322) was performed following the guidelines of the Consolidated Standards of Reporting Trials (CONSORT), with the approval of the Institutional Ethics Committee of the University of Jaén (SEP.20/4.TES) and in compliance with the Declaration of Helsinki. All participants signed an informed consent form.

### 2.2. Participants

The study participants were men and women over 65 years of age with MCI residing in five private institutions that offer care services for the elderly (nursing homes) in the city of Santiago de Cali, Colombia. Recruitment was carried out between January and March 2023 through direct visits in which the project was presented to each institution and the users and their families could decide whether to participate in the study. Each participant was screened with the MMSE to verify that he/she met the required cognitive level.

Participants were included if they met the following criteria: (i) being 65 years of age or older, (ii) voluntary acceptance of participation and signature of informed consent and (iii) having sufficient autonomy to perform physical activities and the ability to follow instructions. Exclusion criteria for this study were: (i) people performing some other exercise program, (ii) contraindicated for performing exercise or physical tests, (iii) medicated with beta-blockers, (iv) diagnosed with moderate or severe cognitive impairment, cancer, pulmonary hypertension, renal failure, heart failure, infected with Human Immunodeficiency Virus (HIV/AIDS), orphan diseases or with neurological disorders and (v) the population that does not accept participation or refuses the use of their data for the research.

### 2.3. Procedures

Randomization was carried out systematically using Epidat 4.2 software (Xunta de Galicia, Consellería de Sanidade-Servizo Galego de Saúde, Spain); additionally, to guarantee blinding, different work teams were organized: one for recruitment and admission of participants, another for the execution of the intervention, and another for pre- and post-measurements. Likewise, the analysis of the results was performed by an epidemiologist who was not involved in the previous process and who received the database in a coded form to keep the information confidential and avoid distinction between the control group and the experimental group.

The sample size was determined with a confidence level of 95% and a power of 90%, with an expected improvement of 10 points in the mean of each measurement in the intervention group (IG). The sample size was 120 people, divided into two balanced groups of 60 people each (60 for the control group and 60 for the intervention group). In addition, this value was adjusted to a probable loss rate of 20%, for which 145 persons were randomized.

### 2.4. Outcomes

Sociodemographic data, including sex, age, socioeconomic strata (according to Law 142 of 1994 that establishes the Regime of Domiciliary Public Services in Colombia), education level, occupation and tobacco and alcohol consumption, was collected through surveys. This data obtained through surveys were then cross-verified and confirmed with the participants’ own registration forms, which were submitted upon their admission to the nursing home.

Cognition was assessed in participants using the following validated scales:

General cognition was assessed with the Montreal cognitive assessment/MoCA test, which evaluated executive and visuospatial function, memory, attention, language, abstraction, recall and orientation [21].

Attention and executive functions were assessed through the Trail Making Test part A and part B (TMTA and TMTB, respectively), which consists of visual and timed motor tasks where participants had to connect consecutively numbered circles (TMTA) or alternating circles of numbers and letters (TMTB) [22]. The shorter the time in which the person manages to connect all the circles, the better the level of attention and executive function is considered [23].

For verbal fluency, the Verbal Fluency Test (VFT) was applied; this test is widely used worldwide in neuropsychological evaluation. The instrument consists of asking the patient to name as many animals as possible for one minute, without using superordinate categories, such as fish, or subordinate categories [24].

Selective attention and concentration were assessed with the D2 test. Selective attention focuses on the subject’s ability to quickly discriminate and select a specific visual stimulus (i.e., the letter “d” with two marks) among other randomly appearing distractor stimuli. This is a time-limited test of following instructions that considers the ability to discriminate stimuli. The output scores are divided into total hits (TH), percentage error (%E), total test effectiveness (TE), calculated from the difference between total words processed and errors, and the concentration index (CON), obtained from the difference between TH and commission errors (errors made by mistakenly marking a letter as correct when it should have been omitted). This test is a useful instrument in research, showing a high degree of validity and reliability [25].

Processing speed was assessed through the Digit Symbol Substitution Test (DSST), a paper-and-pencil cognitive test, presented on a single sheet of paper that requires a subject to match symbols to numbers according to a key located at the top of the page. The subject copies the symbol in spaces below a row of numbers. The number of correct symbols within the time allowed, usually 90 to 120 s, constitutes the score [26]. The maximum score is 60.

### 2.5. Intervention

Two groups were defined in this study.

The intervention group (IG) received a HIFT program for 12 weeks with 3 sessions per week, with a duration of 45 min per session. The sessions were led by a professional in sports science. The exercises to be performed were divided into three phases. Warm-up: 10 min of joint mobility, starting with neck flexion-extension, rotation and lateral flexion-inflection while looking at a fixed point. Trunk mobility exercises executing rotations were performed. For the upper limb, flexion, extension, abduction, and adduction, as well as internal and external rotation, grip exercises and pumping exercises were performed. For the lower limb, hip abduction and adduction from a standing position with upper limb support on the table, as well as hip and knee extension, plantar flexion and ankle dorsiflexion were performed.

The main part consisted of 25 min divided into 4 intervals of 4 min each, performed at an intensity of 80–85% of the maximum heart rate. The exercises included a simulation of a bicycle exercise from a seated position with alternating lower limb movement, wall push-up from a standing position, chair squat with upper limb stabilization support and throwing and catching balls against the wall while performing lateral and frontal lunges. Each exercise was performed for 20 to 30 s as intensely or as fast as possible without generating joint impact, followed by a rest period of 10 to 15 s before repeating the exercise.

The return to calm consisted of 10 min of muscle stretching, with an emphasis on the quadriceps, gluteus maximus and gluteus medius, biceps and triceps brachii and gastrocnemius muscles.

The intervention was standardized and conducted at the group level, with subgroups of 10 people to facilitate logistics and heart rate control using heart rate monitors. Each assistant was assigned a maximum of 2 participants for supervision during the training phase and rest phase, ensuring the necessary intensity was reached.

During the first two weeks, an exercise adaptation protocol was carried out. For this, in the main phase, aerobic training was performed with an initial intensity of 50% to 60% of the maximum heart rate. After the third week, the exercises were divided into four 4 min intervals at an intensity of 80–90% of the maximum heart rate followed by active rest intervals of 3 min at 40–60% of the maximum heart rate. From the fourth week onwards, the exercises continued with the same distribution, but the intensity increased to 85–95% of the maximum heart rate followed by active rest intervals of 3 min at 50–70% of the maximum heart rate.

On the other hand, the control group (CG) participated in the execution of manual activities, such as painting mandalas on paper and decorating picture frames. These activities were directed by occupational therapists and supported logistically by nursing assistants. The CG engaged in these activities for 12 weeks, with a frequency of 3 sessions per week and a duration of 45 min each. Additionally, they were provided with the PAHO physical activity recommendations guide, which had been prepared by professionals in sports science.

### 2.6. Statistical Analysis

The population was characterized according to its socio-demographic conditions, differentiated by groups (CG and IG). Qualitative variables were presented with frequency and percentage in each category. Given the normal distribution of the cognitive outcome variables (Kolmogorov–Smirnov test *p* > 0.05), the data were presented with the mean value and standard deviation (SD). To ensure equality between groups in the initial conditions, chi-square and Student’s *t*-test statistical tests were applied.

Intra-group analysis was performed with a paired *t*-test to evaluate changes in each cognitive test before and after the intervention. The effect size was measured using Cohen’s d, where a value of ≤0.2 indicates no effect, >0.2 and ≤0.5 indicates a small effect, >0.5 and ≤0.8 indicates a medium effect and >0.8 indicates a large effect [27].

For the analysis of the comparison between IG and CG at the end of the intervention, the ANOVA test and the mixed ANOVA analysis were performed, with the factor between groups being participation in or absence from the HIFT program. Each dependent variable was evaluated with possible group-by-time interactions to determine if significant differences exist between the groups over time. The within-group factor was time, and the effect size was evaluated using eta-squared (*η*^2^).

Finally, all the dependent variables of cognition were integrated into a multiple regression model adjusted for the covariates sex, age, initial MMSE level and education level. For all statistical hypothesis tests, a significance level of 0.05 and a confidence level of 95% were established and analyzed using the Stata 14.0 statistical package (STATA Corporation, College Station, TX, USA).

## 3. Results

**Figure 1:**
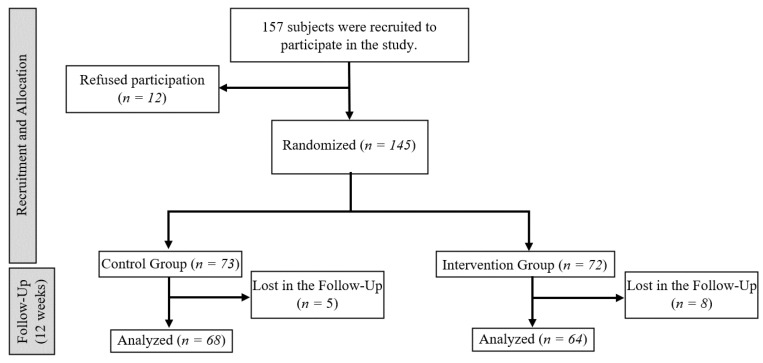
CONSORT flow diagram showing participant selection and allocation.

From a total of 224 elderly residents in the institutions who were eligible, 145 were randomized and allocated to the CG (*n* = 73) and the IG (*n* = 72). During the follow-up, 13 persons dropped out of the study (5 in the CG and 8 in the IG). Finally, 132 patients participated in the study; refer to Figure 1.

The sociodemographic characteristics (Table 1) show a participation rate of 59.8% females and 40.2% males in the population. They belonged to a middle socioeconomic stratum (36.4%), completed secondary education (53.0%) or professional education (28.8%), worked as housewives (41.7%) or were retired (31.8%); they did not use tobacco (95.5%) or consume alcohol (70.5%) and had an average age of 77.15 ± 7.67 years and an MMSE of 21.54 ± 1.42. None of the variables measured at baseline showed significant differences between the CG and IG (*p* > 0.05), which makes the intervention and post-intervention comparison feasible. Additionally, no adverse events were reported during the course of the investigation.

### 3.1. General Cognition

Cognitive impairment measured on the MoCA scale initially showed a mean score of 21.53 ± 1.18 for the CG and 21.63 ± 1.53 for the IG (*p* = 0.687). Post-intervention, a mean difference of 0.902 was found (CG 21.68 ± 1.27 vs. IG 22.58 ± 1.41), which was statistically significant with a medium effect size (*p* < 0.001, Cohen’s *d* = −0.671), highlighting higher scores in participants who received the HIFT program. The intra-group analysis paired *t*-test was −5.55 (*p* < 0.001, Cohen’s *d* = 0.694). The group × time interaction analysis was also significant (*F* = 5.87 *p* = 0.016 *η*^2^ = 0.021), which corroborates the effects of the HIFT intervention and its interaction with the measurement time (see Table 2).

### 3.2. Attention

The attention evaluated at the beginning of the research with the TMTA test, obtained time records of 95.44 ± 10.47 and 97.25 ± 12.71 s for the CG and IG, respectively. After the intervention, the measurements showed a decrease in the time taken to execute the test in the IG (85.86 ± 10.72), which demonstrates a significant improvement compared to the initial conditions (*p* < 0.001, mean difference = −0.953 Cohen’s *d* = 0.694). When post-intervention comparison was made between groups, a difference of 8.12 s was observed, which proves that the intervention with HIFT favors attention and speed with a medium effect (*p* < 0.001, Cohen’s *d* = 0.771). The ANCOVA analysis corroborated the group differences as a function of time (*F* = 13.24, *p* < 0.001, *η*^2^ = 0.044)

### 3.3. Executive Functions

The executive functions assessed through the TMTB at baseline showed time records of 204.25 ± 38.60 for CG and 199.33 ± 29.09 for IG (*p* = 0.412). Once the intervention ended, the means of both groups decreased (CG = 199.49 ± 41.91 and IG = 186.83 ± 24.12) evidencing significant changes within each group, but with a small effect size in CG (*p* = 0.027, mean difference = 5.05, Cohen’s *d* = 0.274) and large effect size in IG (*p* < 0.001, mean difference = 12.5, Cohen’s *d* = 0.880). Consequently, the group × time analysis of covariance was not significant (*F* = 0.834 *p* = 0.36 *η*^2^ = 0.003). The intergroup comparison of the final measurements showed that despite the fact that both showed improvement over time, the intervention with HIFT had a better outcome, but the effect size of this difference was small (mean difference between groups = 12.65, *p* = 0.037, Cohen’s *d* = 0.367).

### 3.4. Verbal Fluency

The verbal fluency assessment through the VFT at baseline showed similar scores (CG = 20.19 ± 3.08 and IG = 19.92 ± 3.18; *p* = 0.622); after 12 weeks of intervention, significant differences were observed between the groups, evidencing the influence of HIFT in the increase of word registration in one minute and a large effect size (CG 22.2 ± 3.08; IG = 25.8 ± 2.43, mean diff = 3.574, *p* < 0.001, Cohen’s *d* = 1.284). The pre- and post-comparison showed that the IG achieved a significant improvement in verbal fluency and its effect size is large (*p* < 0.001, mean difference = −5.844, Cohen’s *d* = −2.366).

Considering the mixed variance group × time, it was possible to establish that significant differences exist between the groups as a function of time, showing changes in the IG that favor processing speed (*F* = 27.8, *p* < 0.001, *η*^2^ = 0.065).

### 3.5. Selective Attention and Concentration

Post-intervention inter-group analysis of the D2 test percentile score (CG = 53.97 ± 14.2 and IG = 51.64 ± 12.1) showed neither significant differences (mean difference = 2.330, *p* = 0.316, Cohen’s *d* = 0.174) nor intra-group analysis of the IG (mean difference = −3.516, *p* = 0.095, Cohen’s *d* = −0. 212). However, when analyzing the individual components of the D2, an increase in the TH with a large effect size (mean difference = −82.093, Cohen’s *d* = −2.601), a decrease in the %E (mean difference = −8.73, *p* < 0.001) with large effect size (Cohen’s *d* = 1.30) and an improvement in the CON (mean difference = −84.09, *p* < 0.001, Cohen’s *d* = 2.53) can be observed in the intra-group comparison of the IG. Inter-group analyses for these variables also prove post-intervention differences in favor of the HIFT program (*p* < 0.001).

In the mixed variance analysis, no group × time interaction was observed (*F* = 0.286, *p* = 0.593, *η*^2^ = 0.001), which indicates that neither the changes in time nor the group were significantly related for the d2 percentile, the total number of responses (*F* = 0.112, *p* = 0.738, *η*^2^ = 0.000) or the total effectiveness of the test (*F* = 0.468, *p* = 0.494, *η*^2^ = 0.002), although it did show an interaction for the TH (*F* = 254, *p* < 0.001, *η*^2^ = 0.300), the %E (*F* = 34.98, *p* < 0.001, *η*^2^ = 0.107) and the CON (*F* = 254.7, *p* < 0.001, *η*^2^ = 0.302).

### 3.6. Processing Speed

Processing speed through DSST score (CG = 48.22 ± 7.56 and IG = 44.77 ± 9.69) did not show significant changes between groups after the intervention (mean difference between groups = 3.455, *p* = 0.24, Cohen’s *d* = 0.399). Considering the mixed variance group × time, it could be determined that there were neither significant differences between groups as a function of time (*F* = 0.069, *p* = 0.793, *η*^2^ = 0.000) nor significant interaction found (*F* = 0.201, *p* < 0.655, *η*^2^ = 0.001).

The multivariate statistical analysis (MANCOVA) integrated all the cognitive variables (Moca, TMTA TMTB, d2, VFT and DSST) and found an interaction with the education level (*p* = 0.026) that favored those with higher levels of education. Adjustment for all sociodemographic variables was performed, which allowed establishing that the effects of the intervention evidenced in all cognitive variables are maintained independently of sex, age and MMSE level (*F* = 62.922, *p* < 0.001, Wilks’ λ = 0.133) (Table 3).

## 4. Discussion

The objective of this study was to determine the effect of a HIFT program on cognitive functions in a Colombian older adult population with MCI. Among the main findings, it was observed that HIFT is an effective strategy to improve general cognition, psychomotor speed and attention, executive functions and verbal fluency, although no significant differences were observed in concentration or information processing speed. Together, these findings corroborate the importance of physical exercise for the brain health of older adults. Considering that cognitive impairment and dementia have become a major burden for health systems, families and the community, in addition to the large financial costs it entails [28,29], this study is of great value, since it proposes new methods of early treatment of dementia to prevent the loss and even improve cognitive performance and quality of life of older adults.

The regular practice of a physical activity is being recognized as a highly protective factor of the cognitive functions [30] and for its neuroprotective effects on brain regions that are vulnerable to neurodegeneration, including the hippocampus and temporal and frontal regions [31,32,33]. Our findings revealed that a 12-week HIFT program improved general cognition in older adults with MCI; similarly, a study conducted on people with dementia in Nigeria [34] showed that a circuit training program is effective in improving, developing and training cognition [35]. Likewise, a randomized trial showed that a high-intensity strength training program, performed for four months significantly improved global cognitive function and maintained the benefits for eighteen months [35].

In relation to the processing speed evaluated with the DSST, no significant improvements were obtained. Consistent with these results, Zhu et al. [36] found that there was no correlation between the physical activity program and processing speed. A systematic review conducted on 809 people receiving interventions based on rhythmic physical activity concluded that the longer the intervention (>13 weeks) the better the effects on cognition [37]. This could explain the lack of significant differences in processing speed since the duration of the study was 12 weeks. It should be highlighted that, although there is evidence of the benefits of HIFT on cognition, due to the heterogeneity of the intervention protocols, it is still necessary to generate more research to determine the dose-response that guarantees the effectiveness of the interventions in relation to all the components of cognition [20].

In this research, attention and executive functions were evaluated through the TMTA and TMTB, respectively. The results showed significant improvements for both tests; however, selective attention and concentration evaluated with the D2 did not show significant differences. This dissimilarity could be explained by the fact that in the D2, attention is not considered as a single aptitude; for this reason, components such as processing speed, precision, stability, fatigue and the effectiveness of attentional inhibition are considered [25]. For the executive functions evaluated with the TMTB, significant changes were found for both CG and IG, with a small effect size for the CG (Cohen’s *d* = 0.274) and a large effect size for the IG (Cohen’s *d* = 0.880). This could be explained since therapies based on visual arts, such as the one proposed in this study in the control group, have been shown to be effective in improving cognitive functions [38,39]. Similarly, with the practice of physical exercise a stronger connectivity can be achieved between the amygdala and the medial temporal gyrus, inferior frontal gyrus, postcentral gyrus and hippocampus, regions that are related to memory and executive functions [40,41]. From these findings, it can be suggested that although it is common to prescribe activities involved with the arts and physical exercise in people with dementia [42], therapies that include physical exercise may be more effective.

Verbal fluency was another variable that showed significant improvements, which is consistent with a study conducted with postmenopausal women where improvements in verbal fluency were found after 12 weeks of Pilates training [43]. Similarly, a systematic review with meta-analysis suggests that physical exercise can produce improvements in verbal fluency in older adults with MCI [44]. It is possible that verbal fluency may be responsive to improvements from physical exercise interventions, especially aerobic exercise, due to the positive selective impact of this type of intervention on the frontal and prefrontal regions of the brain.

Likewise, high-intensity physical exercise has been shown to be more beneficial for brain health than moderate-intensity exercise because, although the physical exercise of different intensities has improvements in different aspects related to brain health, it is vigorous intensity exercise that has the greatest effects on acute levels of circulating brain-derived neurotrophic factor and corticospinal excitability [45,46]. Furthermore, it improves neural plasticity of the hippocampus [47,48], facilitates inhibitory control and its underlying neuro-electrical activation [49], improves brain activation during memory retrieval [50], decreases oxidative stress and anxiety levels and increases antioxidants capacity as a protective system against neuronal damage [51]. In addition, a high-intensity interval training session has a three times shorter duration than a continuous training session of low or moderate intensity [52], which implies a lower time cost while maximizing the beneficial effects at cardiovascular, metabolic and systemic levels [53].

Finally, this study had some limitations. First, the effects of HIFT were evaluated only in the short term, and the intervention time was short. Second, it was conducted only with adults living in the city of Santiago de Cali, Colombia; for this reason, the findings cannot be generalized to other populations. Third, it is important to develop more research that evaluates the effects of HIFT on cognition in older adults with MCI in the long term. And fourth, the lack of consideration for the co-occurrence of other medical conditions and the pharmacological treatments of the patients, apart from those mentioned in the exclusion criteria, could have potentially influenced the results. Among the strengths of this study, we can find its low attrition rate, in addition to its large sample size and its randomized, single-blinded, controlled trial design.

## 5. Conclusions

The present study, developed in Colombian older adults with MCI, demonstrates that a 12-week HIFT program with a frequency of 3 times per week has beneficial effects on general cognition, attention, executive functions and verbal fluency.

Considering that the population of older adults continues to grow and that the prevalence of dementia is increasing, it is important to generate strategies such as the one presented in this study. Similarly, further research on the effects of HIFT is needed to determine the most appropriate dose-response to improve cognition and its different components. In light of these considerations, it is imperative that future research endeavors concentrate on providing comprehensive descriptions of training variables, including volume, time and frequency. Moreover, conducting comparative studies between various training protocols will enable a thorough analysis of the aforementioned dose-response relationships.

## Figures and Tables

**Table 1:** Baseline characteristics of the participants (*n* = 132).

		Total (*n* = 132)	CG (*n* = 68)	IG (*n* = 64)	*p* Value
Age. Mean (SD)		77.2 (7.6)	77.19 (7.7)	77.11 (7.3)	0.951
MMSE. Mean (SD)		21.5 (1.37)	21.56 (1.25)	21.62 (1.59)	0.862
Sex. *n* (%)	Female	79 (59.8)	41 (60.3)	38 (59.4)	0.914
	Male	53 (40.2)	27 (39.7)	26 (40.6)	
Socioeconomic strata. *n* (%)	1	0 (0.0)	0 (0.0)	0 (0.0)	0.731
	2	0 (0.0)	0 (0.0)	0 (0.0)	
	3	45 (34.1)	26 (38.2)	19 (29.7)	
	4	48 (36.4)	23 (33.8)	25 (39.1)	
	5	22 (16.7)	10 (14.7)	12 (18.8)	
	6	17 (12.9)	9 (13.2)	8 (12.5)	
Education level. *n* (%)	Elementary school	21 (15.9)	12 (17.6)	9 (14.1)	0.862
	High school	70 (53.0)	35 (51.5)	35 (54.7)	
	College	38 (28.8)	20 (29.4)	18 (28.1)	
	Postgraduate	3 (2.3)	1 (1.5)	2 (3.1)	
Occupation. *n* (%)	Housewife	55 (41.7)	35 (51.5)	20 (31.3)	0.821
	Businessman	5 (3.8)	3 (4.4)	2 (3.1)	
	Self-employed	30 (22.7)	14 (20.6)	16 (25.0)	
	Employed	42 (31.8)	16 (23.5)	26 (40.6)	
Tobacco consumption. *n* (%)	No	126 (95.5)	63 (92.6)	63 (98.4)	0.115
	Yes	6 (4.5)	5 (7.4)	1 (1.6)	
Alcohol consumption. *n* (%)	No	93 (70.5)	46 (67.6)	47 (73.4)	0.466
	Yes	39 (29.5)	22 (32.4)	17 (26.6)	

SD: standard deviation; MMSE: mini-mental status examination; CG: control group; IG: intervention group; *n*: frequency.

**Table 2:** Effects of the HIFT program over the general cognition, psychomotor attention, attention and executive functions, verbal fluency and processing speed.

Test Mean (SD)	Pre			Post			Group			Time			Group × Time		
	CG	IG	*p* Value	CG	IG	*p* Value	*F*	*p* Value	*η* ^2^	*F*	*p* Value	*η* ^2^	*F*	*p* Value	*η* ^2^
MoCA TEST.	21.53 (1.18)	21.63 (1.53)	0.687	21.68 (1.27)	22.58 (1.41)	<0.001 **	8.98	0.003 *	0.031	10.93	0.001 *	0.038	5.87	0.016 *	0.021
TMTA	95.44 (10.47)	97.25 (12.71)	0.373	93.99 (10.37)	85.86 (10.72)	<0.001 **	5.35	0.021 *	0.018	22.13	<0.001 **	0.074	13.24	<0.001 **	0.044
TMTB	204.25 (38.60)	199.33 (29.09)	0.412	199.49 (41.91)	186.83 (24.12)	0.037 *	3.067	0.039 *	0.016	4155	0.043	0.015	0.834	0.362	0.003
VFT	20.19 (3.08)	19.92 (3.18)	0.622	22.2 (3.08)	25.8 (2.43)	<0.001 **	20.5	<0.001 **	0.048	115.6	<0.001 **	0.273	27.8	<0.001 **	0.065
D2	48.90 (9.88)	48.13 (10.45)	0.663	53.97 (14.29)	51.64 (12.12)	0.316	1.135	0.288	0.004	8.699	0.003 *	0.032	0.286	0.593	0.001
D2-TA	130.71 (17.78)	128.53 (16.02)	0.463	111.99 (19.34)	210.58 (41.56)	<0.001 **	20.8	<0.001 **	0.275	100	<0.001 **	0.118	254	<0.001 **	0.300
D2-%E	6.69 (2.30)	6.80 (1.92)	0.767	9.05 (3.38)	5.37 (2.55)	<0.001 **	31.05	<0.001 **	0.095	02.07	0.151	0.006	34.98	<0.001 **	0.107
D2-TOT	338.38 (51.86)	335.23 (51.74)	0.728	347.47 (67.37)	354.03 (57.74)	0.55	0.058	0.810	0.000	3.861	0.050	0.015	0.468	0.494	0.002
D2-CON	122.29 (18.32)	120.28 (15.93)	0.503	101.40 (20.53)	204.33 (43.39)	<0.001 **	235.5	<0.001 **	0.280	92.2	<0.001 **	0.109	254.7	<0.001 **	0.302
DSST	48.03 (7.63)	45.50 (8.61)	0.076	48.22 (7.56)	44.77 (9.69)	0.24	8.3808	0.004 *	0.031	0.069	0.793	0.000	0.201	0.655	0.001

MoCA: Montreal cognitive assessment; TMTA: Trail Making Test A; TMTB: Trail Making Test B; VFT: Verbal Fluency Test; %E: percentage of error; CON: concentration index; CG: control group; IG: intervention group; DSST: Digit Symbol Substitution Test. *: *p* value < 0.05; **: *p* values < 0.001.

**Table 3:** MANCOVA for MoCA, TMTA TMTB, d2, VFT and DSST for evaluation of interaction with MMSE, group assigned, education level, sex and age.

Model	Wilks’ λ	*F*	*p* Value
GROUP	0.133	62.922	<0.001 **
EDUCATION LEVEL	0.670	1.379	0.087
SEX	0.938	0.631	0.796
GROUP × EDUCATION LEVEL	0.635	1.580	0.026 *
GROUP × SEX	0.932	0.710	0.730
EDUCATION LEVEL × SEX	0.906	0.493	0.976
GROUP × EDUCATION LEVEL × SEX	0.835	0.912	0.579
AGE	0.746	3.285	<0.001 **
MMSE	0.698	4.174	<0.001 **

MMSE: mini-mental status examination. *: *p* value < 0.05; **: *p* values < 0.001.

## Data Availability

The data presented in this study are available on request from the corresponding author. The data are not publicly available because, due to the sensitive nature of the questions asked in this study, participants were assured raw data would remain confidential and would not be shared.
